# Representational change is integral to reasoning

**DOI:** 10.1098/rsta.2022.0052

**Published:** 2023-07-24

**Authors:** Alan Bundy, Xue Li

**Affiliations:** School of Informatics, University of Edinburgh, Edinburgh, UK

**Keywords:** automated theory repair, abduction, belief revision, conceptual change, reformation

## Abstract

Reasoning is the derivation of new knowledge from old. The reasoner must represent both the old and new knowledge. This representation will change as reasoning proceeds. This change will not just be the addition of the new knowledge. We claim that the *representation* of the old knowledge will also often change as a side effect of the reasoning process. For instance, the old knowledge may contain errors, be insufficiently detailed or require new concepts to be introduced. Representational change triggered by reasoning is a common feature of human reasoning but it has been neglected both in Cognitive Science and Artificial Intelligence. We aim to put that right. We exemplify this claim by analysing Imre Lakatos’s rational reconstruction of the evolution of mathematical methodology. We then describe the abduction, belief revision and conceptual change (ABC) theory repair system, which can automate such representational change. We further claim that the ABC system has a diverse range of applications to successfully repair faulty representations.

This article is part of a discussion meeting issue ‘Cognitive artificial intelligence’.

## 1. Lakatos’ proof and refutations

In his book [1], Imre Lakatos illustrates the evolution of mathematical methodology via a rational reconstruction of Euler’s ‘Theorem’ that in a polyhedron V−E+F=2, where V is the number of vertices, F is the number of faces and E is the number of edges. For instance, in a cube V=8, F=6 and E=12.

The setting is a classroom of incredibly bright students whose teacher leads a Socratic dialogue in which the students echo the positions of various prominent mathematicians during the history of this ‘Theorem’.

We have placed scare quotes around ‘Theorem’ because it rapidly becomes apparent that it has a wide variety of counterexamples. The evolution of mathematical methodology is illustrated by the different ways in which these counterexamples are regarded and the attempts to rescue the ‘Theorem’ in some form.

### (a) Cauchy’s ‘proof’ and some counterexamples to it

**Figure 1.**
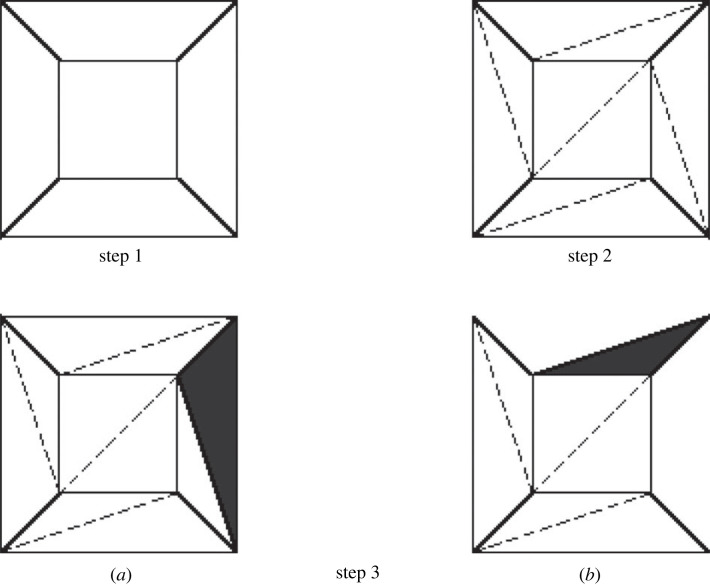
Cauchy’s ‘Proof’ of Euler’s ‘Theorem’. (Redrawn by Predrag Janičić from Lakatos [1]). This ‘proof’ consists of a procedure. In Step 1, a face is removed from the polyhedron and it is stretched onto the plane. In Step 2, each face is triangulated. In Step 3, these triangles are successively removed until only one remains. There are two different cases of triangle removal, labelled (*a*) and (*b*). The last triangle has three vertices, three edges and one face, so V−E+F=1. It is argued that this invariant is preserved by each step, except the first, which removes one face. So, working backwards, it has been shown that in the original polyhedron, V−E+F=2. QED.

Lakatos’ book starts with a ‘proof’, due to Cauchy, illustrated by figure 1.

**Figure 2.**
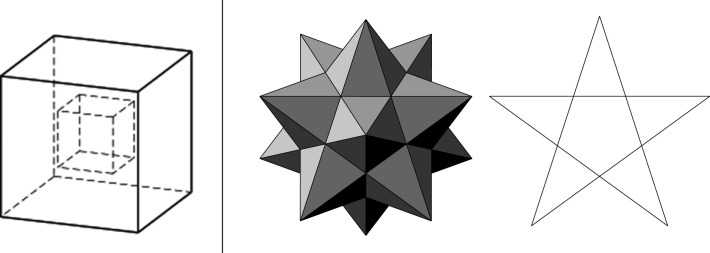
The hollow cube, Kepler’s star polyhedron and pentagram (redrawn by Predrag Janičić from Lakatos [1]). The hollow cube has a cube-shaped hole in it, so the number of vertices, faces and edges is doubled: V−E+F=4. The faces of Keplar’s star polyhedron are intersecting pentagrams. There are 12 of these faces, making 30 edges and 12 vertices, so V−E+F=−6. Note that, in neither counterexample, is it possible to remove one of their faces and stretch the remaining polyhedron flat on the plane.

We have put scare quotes around ‘proof’ because Cauchy’s ‘Proof’ is no more a proof than Euler’s ‘Theorem’ is a theorem. It is claimed that this same procedure can be carried out on any polyhedron. But this claim has been shown to be false. Lakatos’ students soon come up with counterexamples. Two of these are depicted in figure 2.

### (b) The changing definition of polyhedron

The students, however, are able to rescue these counterexamples and turn them into examples. They did this by choosing an appropriate definition of polyhedron or polygon. In the case of the hollow cube, they contrasted a solid structure with a plate structure.

Solid structure: A polyhedron is a *solid* whose surface consists of polygonal faces.
Plate structure: A polyhedron is a *surface* consisting of a system of polygons.

In the case of the *plate* structure, the hollow cube becomes one cube nested within another. For each one V−E+F=2.

Intersecting edges: A polygon is a system of edges arranged in such a way that exactly two edges meet at each vertex.
Non-intersecting edges: A polygon is a system of edges arranged in such a way that exactly two edges meet at each vertex *and the edges have no points in common except the vertices.*

The second definition excludes edges that intersect, so the pentagram is ruled out as a polygon. If, on the other hand, we interpret the triangles as the faces, then there are 32 of them, with 90 edges and 60 vertices, so V−E+F=60−90+32=2.

Lakatos calls this tactic of rejecting a counterexample via a change of definition, *monster barring*.

Now we are confronted by a conundrum:

*How was it possible for Euler to state a conjecture about polyhedra and for Cauchy to claim to have proved that conjecture, if neither had a formal definition of polyhedra?*

The answer is that both were generalizing from a finite set of examples. They knew about the five Platonic solids of a tetrahedron, cube, octahedron, dodecahedron, icosahedron and a few more. Euler’s conjecture and Cauchy’s proof both worked for them. There were others, however, for which whether it worked or not depended on definitions that they had not formulated.

We claim that this state of affairs is commonplace. Despite it imposing a strict discipline of formal definitions, representational change can even arise in modern mathematics. For instance, Robinson’s non-standard analysis [2] has extended the concept of ‘number’ to include infinite and infinitesimal numbers, and intuitionistic logic has divided the concept of ‘proof’ into ‘classical’ and ‘constructive’.

Reasoning based only on examples is a frequent cause of computer failures. A program may run successfully on a finite set of test examples then, but later encounter so-called, ‘edge cases’, on which they fail.

Formal verification of computer programs can avoid this problem because it proves the correctness of the program for the, potentially infinite, set of all cases. It requires, though, a formal logical definition of *all cases* and this is still error prone since it is a non-trivial task to formulate such a definition.

This motivated us to attempt to automate the repair of faulty logical theories. This is not just a matter of enlarging or reducing the axioms of a theory. It also entails refining the *language* in which they are expressed. We saw such language refinement in the case of polyhedra in the debate over whether they were solid or plate structured and over whether a polygon’s edges could intersect. In this spirit, we developed the *ABC theory repair system* [3,4].

## 2. The ABC theory repair system

The ABC system takes a faulty theory T and a preferred structure PS. It uses PS and inference on T to identify faults in T. It then applies repair operations to T in an attempt to correct the faults. If it terminates, it outputs all fault-free theories that it can find.

Theories are expressed in the *Datalog* language. See §2a for details. The preferred structure PS is a pair ⟨T(PS),F(PS)⟩ of sets of ground assertions representing observations of the environment. T(PS) represents ground assertions that *are* observed and F(PS) represents ground assertions that are observed to be false. Faults are either *insufficiencies*, where something in T(PS) is not predicted or incompatibilities, where something in F(PS) *is* predicted. By predicting a ground assertion we mean that it is a theorem of T. To prove theorems we use selected literal (SL) resolution [5]. See §2b for details.

**Figure 3.**
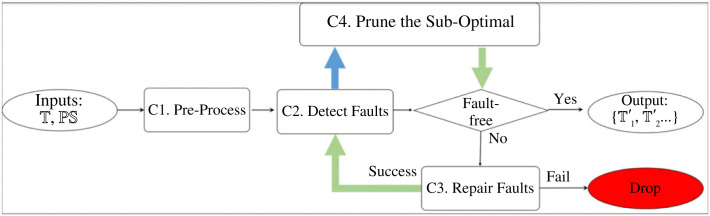
The flowchart of ABC. The green arrows deliver a set of theories one by one to the next process; the blue arrow delivers all faults of one theory as a set. When a faulty theory is not repairable, it will be dropped. Thus, the fault detection and repair generation is recursive until the repair process terminates with fault-free theories or finds no further repairs to apply.

The flowchart of the ABC system is depicted in figure 3. The pre-process C1 reads and rewrites inputs into the internal format for later use. Then in C2, ABC applies SL to T to detect incompatibility and insufficiency faults.

*We have claimed above that the ABC System has a diverse range of applications for successfully repairing faulty representations.*

We evaluate this claim in §5 by presenting a diverse range of applications to which ABC has been successfully applied.

### (a) Datalog theories

Datalog is a logic programming language consisting of Horn clauses in which there are no functions except constants [6]. We use this notation to define a subset of first-order logic that we also call *Datalog*. We choose Datalog because it is decidable under SL and ABC requires^1^ decidability in the detection of faults. We represent formulae in Datalog as clauses in Kowalski normal form, shown in definition 2.1 below.

**Definition 2.1. (Datalog formulae)**

Let the *language* of a Datalog theory T be a triple ⟨P,C,V⟩, where P are the propositions, C are the constants and V are the variables. We will adopt the convention that variables are written in lower case, and constants and predicates start with a capital letter.^2^

A proposition is a formula of the form $P(t_{1},\ldots ,t_{n})$, where $t_{j}\in C\cup V$ for 1≤j≤n, i.e. there are no compound terms. Let R∈P and $Q_{i}\in P$ for 0≤i≤m in T. Datalog clauses are of the four types in definition 2.2, R is called the *head* of the clause and the conjunction of the $Q_{i}$s forms the *body*.

**Definition 2.2.**

Kowalski Form Horn clauses

Implication: $(Q_{1}\wedge \ldots \wedge Q_{m})\;⟹\;R$, where m>0. These usually represent the rules of T.
Assertion:  ⟹ R, i.e. the body is empty. When R contains no variables the assertion is called *ground*. These ground assertions represent the facts of T and the members of T(PS) and F(PS).
Goals: $Q_{1}\wedge \ldots \wedge Q_{m}\;⟹\;$, i.e. the head is empty. These usually arise from the negation of the conjecture to be proved and from subsequent subgoals in a derivation.
Empty Clause:  ⟹ , i.e. both the head and body are empty. This represents false, which is the target of a reductio ad absurdum proof. Deriving it, therefore, represents success in proving a conjecture.

### (b) Selected literal resolution

SL is a complete, reductio ad absurdum proof procedure for first-order logic expressed in clausal form [5]. When restricted to Datalog clauses, such as those defined in definition 2.2, SL is a decision procedure. This means that ABC can decide whether a given conjecture is or is not a theorem of a Datalog theory T.

**Definition 2.3. (A deductive step in SL)**

A deductive step in SL is between a goal clause and either an assertion or a rule, which we will collectively call an *axiom*. A proposition in the goal clause and the head of the axiom must unify, that is it must be possible to instantiate each of them so that they are identical. An instantiation σ replaces variables by constants. We write ϕσ to mean that proposition ϕ is instantiated by substitution σ. The substitution used in an SL step is called *a most general unifier* [8]. We will depict such an SL step as

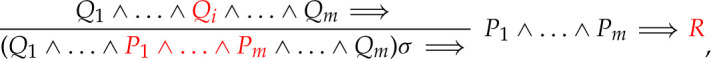

where σ is the most general unifier of $Q_{i}$ and R.

**Definition 2.4. (An SL refutation)**

Because a proof in SL is by reductio ad absurdum, we call it a *refutation*. It consists of a series of SL steps. Each step takes as input the goal clauses produced by the previous step and outputs the goal clauses to be used in the next step. In the final step, one goal proposition remains and an assertion is unified with it to leave the empty goal clause  ⟹ . We can depict such a refutation as follows:

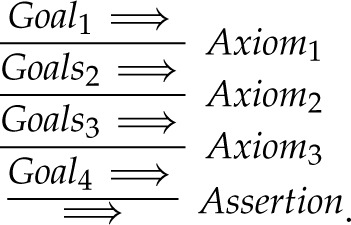

If an SL refutation proves a formula ϕ using the axioms from theory T. We write T⊢ϕ.

### (c) Types of fault

Given a preferred structure PS, a theory T could have two kinds of faults:

Incompatibility: Predictions that arise from the agent’s representation conflict with observations of their environment: ∃ϕ. T⊢ϕ∧ϕ∈F(PS).
Insufficiency: The agent fails to predict observations of its environment: ∃ϕ. T⊬ϕ∧ϕ∈T(PS).

Since SL is decidable for Datalog theories [9], ABC can exhaustively test all members of both T(PS) and F(PS) for theorem-hood. So it can detect all occurrences of insufficiency and incompatibility in a Datalog theory.

### (d) ABC repair operations

An insufficiency may be repaired by unblocking a proof with additional necessary SL steps, while an incompatibility may be repaired by blocking all its proofs, which can be done by breaking one SL step in each of them [3]. ABC repairs faulty theories using 11 *repair operations*. There are five for repairing incompatibilities and six for repairing insufficiencies. These are defined in definitions 2.5 and 2.6.

**Definition 2.5. (Repair operations for incompatibility)**

In the case of incompatibility, the unwanted proof can be blocked by causing any of the SL steps to fail. Suppose the targeted SL step is between a goal, $P(s_{1},\ldots ,s_{n})$, and an axiom, $Body\;⟹\;P(t_{1},\ldots ,t_{n})$, where each $s_{i}$ and $t_{i}$ pair can be unified. Possible repair operations are as follows:

Belief Revision 1: Delete the targeted axiom: $Body\;⟹\;P(t_{1},\ldots ,t_{n})$.
Belief Revision 2: Add an additional precondition to the body of an earlier rule axiom which will become an unprovable subgoal in the unwanted proof.
Reformation 1c: Rename P in the targeted axiom to either a new predicate or a different existing predicate $P^{\prime }$.
Reformation 2c: Increase the arity of all occurrences P in the axioms by adding a new argument. Ensure that the new arguments in the targeted occurrence of P, are not unifiable. In Datalog, this can only be ensured if they are unequal constants at the point of unification.
Reformation 3c: For some i, suppose $s_{i}$ is C. Since $s_{i}$ and $t_{i}$ unify, $t_{i}$ is either C or a variable. Change $t_{i}$ to either a new constant or a different existing constant $C^{\prime }$.

**Definition 2.6. (Repair operations for insufficiency)**

In the case of insufficiency, the wanted but failed proof can be unblocked by causing a currently failing SL step to succeed. Suppose the chosen SL step is between a goal $P(s_{1},\ldots ,s_{m})$ and an axiom $Body\;⟹\;P^{\prime }(t_{1},\ldots ,t_{n})$, where either $P\neq P^{\prime }\;\mathrm{or}\;m\neq n$ or for some i, $s_{i}$ and $t_{i}$ cannot be unified. Possible repair operations are

**Abduction 1:** Add the goal $P(s_{1},\ldots ,s_{m})$ as a new assertion and replace variables with constants.
**Abduction 2:** Add a new rule whose head unifies with the goal $P(s_{1},\ldots ,s_{m})$ by analogizing an existing rule or formalizing a precondition based on a theorem whose arguments overlap with the ones of that goal.
**Abduction 3:** Locate the rule axiom whose precondition created this goal and delete this precondition from the rule.
**Reformation 1s:** Replace $P^{\prime }(t_{1},\ldots ,t_{n})$ in the axiom with $P(s_{1},\ldots ,s_{m})$.
**Reformation 2s:** Suppose m=n but $s_{i}$ and $t_{i}$ are not unifiable. Decrease the arity of all occurrences $P^{\prime }$ by 1 by deleting its ith argument.
**Reformation 3s:** If m=n but $s_{i}$ and $t_{i}$ are not unifiable, then they are unequal constants, say, C and $C^{\prime }$. Either (i) rename all occurrences of $C^{\prime }$ in the axioms to C or (ii) replace the offending occurrence of $C^{\prime }$ in the targeted axiom by a new variable.

Belief Revision 1 is inherited from Gärdenfors [10]. Belief Revision 2 extends this by adding a precondition that will create a subgoal that will be unprovable in the unwanted proof. Similarly, Abduction 1 is inherited from Cox & Pietrzykowski [11] and Abduction 2 extends this by removing a precondition that has given rise to an unprovable subgoal. The various Reformation operations are adapted from Bundy & Mitrovic [7]. They break new ground by evolving the language of the faulty theory, e.g. by creating new predicates and constants. In [7], the different forms of Reformation arose by an exhaustive analysis of ways in which the first-order unification algorithm [8] could defeat or enable the matching of a goal to an axiom in an unwanted proof or a failed but wanted proof, respectively.

We are not claiming that these 11 repair operations are complete. Indeed, we doubt that a claim of completeness could be made of *any* set of theory repair operations. Human ingenuity is unbounded and no one could anticipate all possible forms that theory repair could take. Moreover, the forms of theory repair depend on the kind of logic that a theory is expressed in. In a sorted logic, for instance, manipulations of the type hierarchy offer additional opportunities [12]. In an equational logic, different forms of inference are required, but proofs can still be blocked or unblocked at the application of a matching operation [13]. In a probabilistic logic, the assigned probabilities can be changed [14]. Therefore, we can only make *empirical* claims about the range of applications of the ABC system.

### (e) Related work

In §2d, we briefly discussed the relationship of the ABC repair operations to both belief revision [10] and abduction [11]. There is also a tradition of theory revision in Inductive Logic Programming. In [15], Shapiro presented an algorithm for debugging logic programs. Given a faulty logic program, it recurses through a failed execution of the program using an oracle to check the truth of each sub-routine call. If one of these is false, this process will eventually identify the root cause of the fault, namely a false clause. This clause can then be replaced. [16,17] subsequently built on this work. For instance, de Raedt’s RUTH system used integrity constraints to provide the oracle and repaired faulty programs by a combination of belief revision and abduction.

These theory revision programs focus on the *identification* of the faults. The ABC System uses the preferred structure to do this, by trying to prove assertions from both T(PS) and F(PS). Success in proving an assertion in F(PS) constitutes an incompatibility and failure to prove an assertion in T(PS) constitutes an insufficiency. ABC’s novelty lies, though, in its repair operations, which go beyond belief revision and abduction, to include reformation. ABC does not just add or delete clauses but changes the *language* in which they are expressed. Thus systems such as RUTH and ABC are complementary and could be combined.

## 3. Pruning and ranking ABC repairs

**Figure 4.**
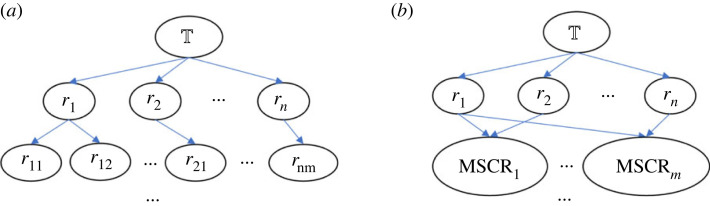
The search space for fault-free theories. (*a*) The Naive search space and (*b*) the reduced search space based on MSCRs. The length of each search branch can be different. By applying all repairs in one search branch, that branch terminates with a fault-free theory or with failure, if no repair is available to fix a detected fault.

In a faulty theory, there can be multiple faults and each fault can have multiple repairs. The basic search is shown in figure 4*a*, where repairs are applied individually and the fault detection and repair generation are recursive until the repair process terminates with fault-free theories or finds no further repairs to apply. So, if ABC outputs any repaired theories they are necessarily fault-free, but that does not make them equally good.

We have, therefore, explored mechanisms for pruning implausible or sub-optimal repairs—or by ranking them. In §3a below, we describe one such mechanism and then mention two others.

### (a) The optimal maximal set of commutative repairs

*Commutative repairs* are the ones that can be applied in any order, for instance, because they repair different parts of a theory to solve different faults. Thus, we refine the naive search method so that it computes only maximal sets of commutatives repairs (MSCRs). As the repairs in an MSCR can be applied together, the search space of fault-free theories using MSCR is reduced dramatically.

Not all repairs are commutative.

(i) It is possible that after applying one repair $r_{1}$, another repair $r_{2}$ will not be needed because $r_{1}$ has also solved the fault which $r_{2}$ targeted.
(ii) On the other hand, $r_{1}$ may make $r_{2}$ inapplicable. For instance, $r_{1}$ may merge predicate mother with predicate mum. Then, if $r_{2}$ would have deleted an axiom of mother, it cannot find it after $r_{1}$’s application.

These are the scenarios where repairs are not commutative because applying them in different orders results in different repaired theories.

The commutation between repairs $r_{1}$ and $r_{2}$ is defined in definition 3.1. T⋅r represents the application of a repair r to a theory T.

**Definition 3.1. (Commutative repairs)**

Two repairs $r_{1}$ and $r_{2}$ are commutative if applying them in different orders to theory T results in the same repaired theory.

3.1 $$T\cdot r_{1}\cdot r_{2}=T\cdot r_{2}\cdot r_{1}.$$

Accordingly, the maximal set of commutative repairs is defined in definition 3.2.

**Definition 3.2. (Maximal set of commutative repairs)**

Given the whole set of all possible repairs R for all detected faults in the theory T, an MSCR M is a maximum set of T’s commutative repairs if they avoid the scenarios illustrated in (i) and (ii). We can formalize this as follows:

3.2 $$\exists r_{m}\in M,\;\forall r\in R\setminus M,\;S(T,r)\wedge S(T,r_{m})\neq \emptyset$$

and

3.3 $$\forall r_{1},r_{2}\in M,\text{ if }r_{1}\neq r_{2},\text{ then }F(T,\;r_{1})\neq F(T,\;r_{2}),$$

where S(T,r)={α|α∈T∧α∉T⋅r} is the scope of a repair r in the theory T, and F(T,r) is the fault in T that a repair r solves.

There could be n MSCRs, where n≥1, and a repair can belong to more than one MSCR. ABC computes all MSCRs and applies each MSCR separately to produce n semi-repaired theories, which will be delivered for the next round of fault detection and repair generation. By applying repairs in an MSCR together, the search space is reduced because the search branches of grouped repairs are merged into one. The comparison of search spaces is drawn in figure 4.

Inspired by the sub-optimal pruning of ABC repairs based on Max-Sat [18], an optimal MSCR is defined as follows:

**Definition 3.3. (Optimal MSCR)**

An MSCR, $M_{1}$, is optimal for the theory T if and only its estimated cost $c(T\;\;M_{1})$ is not bigger than any of the MSCRs of that theory, denoted as $M_{2}$:

3.4 $$\forall M_{2}.\;c(T,\;M_{1})\leq c(T,\;M_{2}),$$

where the estimated cost is

$$c(T,\;M)=|M|+N_{\text{insuff}}(T_{m})+N_{\text{incomp}}(T_{m}),$$

where |M| is the number of repairs in M and $N_{\text{insuff}}(T_{m})$ and $N_{\text{incomp}}(T_{m})$ are the number of insufficiencies and incompatibilities of $T_{m}$, respectively, and where $T_{m}$ is the repaired theory produced by applying all repairs in M to T.

By only taking the optimal MSCRs to the remaining repair process, the search space of fault-free theories is further reduced. This method dramatically saves time and space.

### (b) Other ranking mechanisms

Another ranking mechanism is inspired by Gärdenfors *epistemic entrenchment* [10]. This is defined to represent the informational value of a belief so that it can guide a repair system to change the beliefs of the least informational value while keeping the others untouched. Based on the idea of epistemic entrenchment, the vitality of an axiom or a precondition is defined as scores [19], which rank repaired theories so that the best ones are prioritized at the top. Also, the entrenchment of the theory’s signature^3^ is explored to rank repairs [20].

Another ranking mechanism is based on probability. In a probabilistic logic, a theory can be assigned an overall probability based on the probabilities assigned to its assertions and implications. A probabilistic version of ABC is described in [14].

In future work, we hope to explore the design of extensions to the preferred structure that might distinguish between alternative repairs. Consider, for instance, the use of ABC to diagnose what misconceptions about arithmetic might be the cause of a student’s errors, which we discuss below in §5e. More than one misconception might explain the observed errors. A teacher might set the student an arithmetic problem that would result in different outcomes depending on which misconception the student was suffering from.

## 4. Illustrative example: the black swan

In this section we illustrate the operation of ABC using the black swan theory, which is given in example 4.1. This example was drawn from the belief revision literature [21], where the proposed repair operations are to remove one of the four axioms: A1−A4.

Let the theory in 4.1 be T.


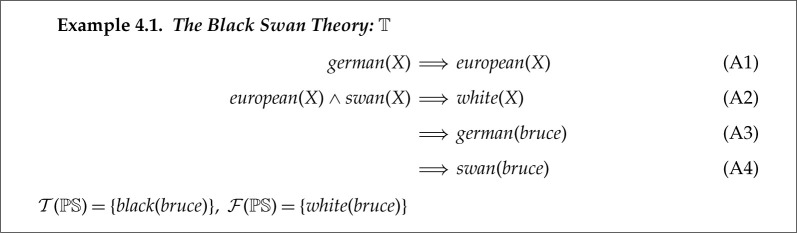


T has both an incompatibility fault and an insufficiency one.

T⊢white(bruce)∧white(bruce)∈F(PS)

and

T⊬black(bruce)∧black(bruce)∈T(PS)

T illustrates a limitation of relying on just belief revision for repairing faulty theories. None of the four axiom removal operations results in what we suggest is the most natural repair to its incompatibility fault. We think this fault arises from the ambiguity of european(X): it could mean ‘X is a European variety’ or ‘X is resident in Europe’. In the first case, since bruce is black then european(bruce) is false, but in the second case it could be true if bruce is resident in Germany, e.g. in a zoo.^4^ ABC’s Reformation 2c repair adds an extra argument to predicate european that enables this distinction.

**Figure 5.**
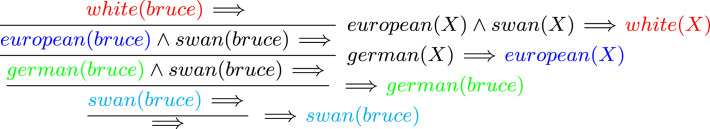
SL resolution steps of the incompatibility. A different colour is used to highlight each pair of unifying propositions.

We will start by repairing this incompatibility fault. To see that T⊢white(bruce), consider the SL proof in figure 5.

The proof in figure 5 can be broken at any of these four coloured unification steps. We will illustrate it being broken at the blue pair, i.e. between european(bruce) and european(X). We choose the repair operation Reformation 2c, which will add a new argument to european to distinguish its two possible meanings. ABC is not able to assign meanings to new constants, so we use abnormal to the instance in the goal clause and normal to that in the axiom.^5^ In this example, humans can interpret abnormal as ‘resident’ and normal as ‘variety’. Note that instances in other rules in T, such as (TA1) are assigned a new variable. The resulting (and desired) repair of T is given by example 4.2 with changes highlighted in red.


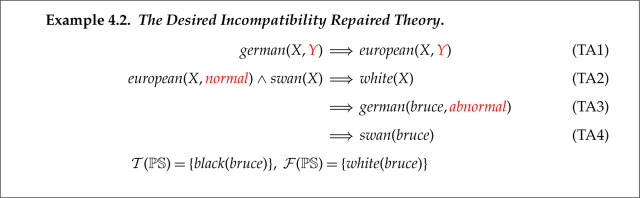


The incompatibility fault has been repaired, as the proof of white(bruce) in figure 5 is now broken.

We now illustrate ABC’s repair of the insufficiency. Its simplest repair is adding the preferred proposition as an axiom directly using Abduction 1. This is illustrated in example 4.3. The required proof of black(bruce) consists of one step between the goal and this new axiom.


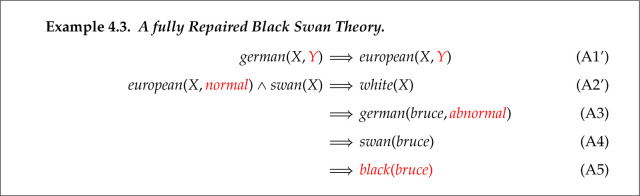


This final repaired theory is fault-free with respect to PS. The repaired theory is generated by combining reformation and abduction, and the solution satisfies the claimed repair postulates.

ABC can find 39 ways to repair theory 4.1 by breaking the proof at different points and by choosing different repair operations to break it. Many of these repairs also support a meaningful interpretation. Our work on mechanisms for preferring one repair over another are described in §3.

## 5. Applications of theory repair

In §2, we claimed that:

*The ABC system has a diverse range of applications in successfully repairing faulty representations.*

Our evidence to support this claim is to present some diverse examples of faulty theories that the ABC system has successfully repaired. Is a claim of this kind *refutable*? Suppose we had found a significant set of applications of theory repair that could *not* be successfully addressed by ABC. This could be interpreted as a refutation of our claim. We did not find such a set. Indeed, each of the applications we attempted was successful. As mentioned in §2d, different logics present both new repair challenges and opportunities. In each logic we have examined, we have been able to make straightforward adaptations of ABC to enable it to successfully address these new repair challenges. In §6, we discuss the example of Lakatos’s monster barring repairs.

### (a) Defeasible reasoning

Defeasible reasoning occurs when a rule is given, but there may be specific exceptions to it. We will call such a rule *defeasible*. In AI, defeasible reasoning has usually been formalized by some kind of *non-monotonic logic* [22]. Logical theories are normally^6^ *monotonic*, i.e. adding extra axioms to a theory increases its set of theorems. In a non-monotonic theory adding a new axiom can sometimes override some applications of a defeasible rule, so that of proofs of some theorems will no longer hold.

We offer an alternative mechanism using only monotonic logical theories, i.e. a monotonic theory containing a faulty rule is repaired into another monotonic theory in which the fault has been eliminated.

The classic example of defeasible reasoning is about tweety the penguin: a non-flying bird. We have formalized both the original faulty theory and its repair in example 5.1.


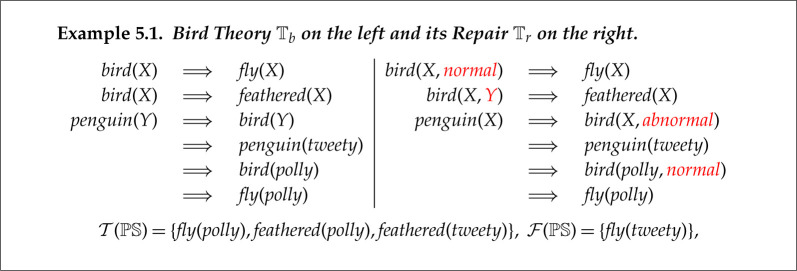


The left hand theory $T_{b}$ has an incompatibility fault: $T_{b}⊢fly(tweety)$ but fly(tweety)∈F(PS). The repair operation Reformation 2c is applied to add an additional argument to bird in all occurrences in theory $T_{r}$ and these are highlighted in red. This argument is given the value normal, abnormal or a variable. This prevents the unification of bird(X,normal) with bird(X,abnormal), which is required to prove fly(tweety). The proofs of the members of T(PS) are unaffected by this repair.

ABC also finds a repair in which Reformation 1c is used to rename bird in the axiom to a new predicate ${bird}^{\prime }$, representing flying birds, where bird now means non-flying ones. To avoid introducing an insufficiency, the axiom bird(X) ⟹ feathered(X) axiom now has to be duplicated for ${bird}^{\prime }$. This is a disadvantage of Reformation 1c over Reformation 2c in this case.

### (b) Modelling virtual bargaining

*Virtual Bargaining* is a term coined by Cognitive Scientist, Nick Chater, to describe the extraordinary ability to cooperate with humans to reach a, sometimes complex, agreement with only minimal channels of communication. It relies on their ability to put themselves in the shoes of their partner to imagine how they will understand these minimal communications [23].

To illustrate virtual bargaining, Misyak *et al.* [23] invent a two-person cooperative game called *bananas and scorpions*. In this game, the human players need to guess or adjust the winning strategy based only on the others’ game moves. The two players are the sender and the receiver. There are three boxes of two kinds: the helpful (containing bananas) and the harmful (containing scorpions). The sender knows all the boxes’ contents and marks one of them to guide the receiver to choose a helpful box by marking only one box. On the other hand, the receiver only knows the number of each type of box, and which box is marked, and then aims to select as many helpful boxes as possible.

**Figure 6.**
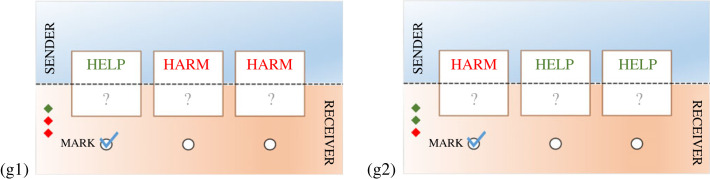
Two rounds of the scorpions and bananas game. The sender’s view is at the top and the receiver’s at the bottom. The sender knows what is in each box, the receiver does not. The receiver does, however, know how many helpful and harmful boxes there are, indicated by the red or green diamonds at the bottom left. The sender marks one box with a tick and the receiver can see what was marked. The receiver can open as many boxes as they like but must avoid harmful ones while opening as many helpful ones as possible.

Two rounds of the game are illustrated in figure 6. In the first round (g1), only box b1 is helpful and boxes b2 and b3 are harmful while the opposite is true in the second round (g2): b2 and b3 are helpful and b1 is harmful.

Given this limited bandwidth, each player has to imagine what the other is thinking and plan their play. In situation (g1), the sender has marked the only box marked ‘Help’. This is a natural strategy to adopt. It is akin to pointing at the box you want to have opened. But in the situation (g2), the strategy is less obvious. The sender could mark one of the two ‘Help’ boxes, but cannot mark both, as only one tick is allowed. The receiver could then open this box, but this is a sub-optimal outcome, as the best outcome would be to open both ‘Help’ boxes. Bearing this in mind, the sender has changed strategy to mark the single ‘Harm’ box, intending the receiver to open both ‘Help’ boxes. Remarkably, the human players of the game frequently and spontaneously adopted this strategy, thus confirming their ability to do virtual bargaining. They did not first have to experiment to see which strategy was being used.

In [24], our research group modelled this process. Our Datalog theories were logic programs that the sender and receiver would invent and the receiver could then use to select which boxes to open. The starting theory T contains an insufficiency, which is easily repaired to solve g1. However, it would have failed completely on g2, because it would have opened a harmful box and would have failed to open either of the helpful ones.

The key rule in T was

5.1 mark(X,Y) ⟹ select(X,Y),

which can be interpreted as ‘select the marked box’, where mark(X,Y) means ‘in game X mark box Y’ and select(X,Y) means ‘in game X select box Y’. The preferred structure was

$$T(PS)=\{select(g_{1},b_{\text{green}}),\;select(g_{2},b_{\text{green}1}),\;select(g_{2},b_{\text{green}2})\}$$

and

$$F(PS)=\{select(g_{1},b_{\text{red}1}),select(g_{1},b_{\text{red}2}),\;select(g_{2},b_{\text{red}})\}.$$

Using the red and green diamonds, this reflects the receiver’s knowledge of the two games. In $g_{1}$, $b_{\text{green}}$ stands for whichever box is known to be helpful and $b_{\text{red1}}$ and $b_{\text{red}2}$ the two boxes known to be harmful. In $g_{2}$, it is $b_{\text{green}1}$ and $b_{\text{green}2}$ that are known to be helpful and $b_{\text{red}}$ that is harmful. Note that we must not assume that the receiver knows which actual boxes these correspond to, e.g. the receiver does not initially know that in $g_{1}$, $b_{\text{green}}$ will turn out to be $b_{1}$, the marked box.

The insufficiency in $g_{1}$ is that $T⊬select(g_{1},b_{\text{green}})$. The proof fails because $mark(g_{1},b_{\text{green}})$ does not unify with $mark(g_{1},b_{1})$, where $b_{1}$ is the leftmost and marked box. This insufficiency is easily fixed using Reformation 3s, by replacing $b_{1}$ with $b_{\text{green}}$ in axiom mark(g1,b1).

In $g_{2}$, T has both incompatibility and insufficiency faults, namely:

$$\begin{array}{rl} T⊢select(g_{2},b_{\text{red}}) & \;\wedge \;select(g_{2},b_{\text{red}})\in F(PS)\\ T⊬select(g_{2},b_{\text{green1}}) & \;\wedge \;select(g_{2},b_{\text{green1}})\in T(PS)\\ T⊬select(g_{2},b_{\text{green2}}) & \;\wedge \;select(g_{2},b_{\text{green2}})\in T(PS) \end{array}$$

The simple repair that worked for $g_{1}$ will not work for $g_{2}$ because both $select(g_{2},b_{\text{green1}})$ and $select(g_{2},b_{\text{green2}})$ have to be proved and $b_{1}$ cannot be merged with both $b_{\text{green1}}$ and $b_{\text{green2}}$ because they are unequal.

Instead, ABC repairs the faulty T using the operation Belief Revision 2, namely adding extra preconditions to rule 5.1. This rule is duplicated and the two new rules are given different preconditions to distinguish the two situations: when there are more harmful than helpful boxes or the other way around. These two new rules are

5.2 hp<hm∧mark(X,Y) ⟹ select(X,Y)

and

5.3 hm<hp∧Y≠Z∧mark(X,Y) ⟹ select(X,Z),

where hm is the number of harmful boxes and hp the number of helpful ones in the game. Using rule 5.3, the repaired theory suggests opening all boxes that are not marked.^7^ With these new rules, the repaired theory is able to correct the incompatibility and both the insufficiencies.

### (c) Root cause analysis

Root cause analysis (RCA) diagnoses faults in a network system using system logs,^8^ where single causes can trigger multiple failures. Taking the input theory, containing the information from system logs and domain rules, ABC can detect missing information (MI) that is essential to cause failures and then suggests repairs that fix root causes [25].

**Figure 7.**
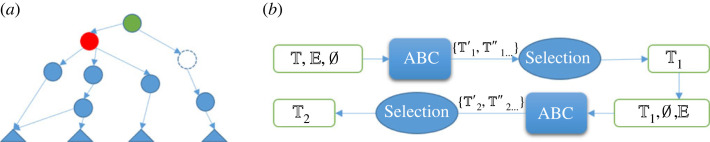
ABC in root cause analysis. (*a*) Failed RCA due to MI. Triangles are propositions describing system failures; circle nodes are axioms or theorems representing system behaviours; an arrow starts from a behaviour’s representation to its logical consequences; the dashed node corresponds to the axiom that should be added to represent MI, which is not in the original theory describing the network system; (*b*) ABC’s flow of two-step RCA. RCA’s inputs are (i) theory T; (ii) the observed system failures as a set of assertions E. RCA’s output is the repaired theory $T_{2}$ where the root cause is addressed. Here ABC’s inputs are a theory, T(PS) and F(PS) in turn: in the first step T(PS)=E, F(PS)=∅; ABC outputs potential repairs $\left\{T_{1}^{\prime },T_{1}^{\prime \prime }\cdots \right\}$, from which the selected $T_{1}$ is the input theory of the second step, where T(PS)=∅,F(PS)=E.

Figure 7*a* shows the damage caused by MI in RCA. All nodes are explicitly in the theory, which models the software system, except the dashed node.^9^ Due to MI, the green node will not be diagnosed as the root cause of all four failures, as it should be. Figure 7*b* depicts ABC’s workflow in RCA, where the faulty T, which lacks MI, is repaired into $T_{1}$ to cover the previous missed information and then $T_{2}$ includes repairs that fix root causes.

The example given by Li & Bundy [25] is about microservices in a network system. *In the first step of RCA*, ABC detects two insufficiencies in the original faulty theory, because the theory fails to predict two microservices failures. The first insufficiency is caused by the MI of a microservice session: that id is built on a full board d1. Thus, the repair is to add the corresponding axiom (5.4) (in green). The other insufficiency is cased by the mismatch between the predicate microservice in (5.5) and the predicate ms in (5.6) (in red). The repair is to merge them, e.g. rename ms to microservice. In the second step of RCA, ABC changes the full board to not being full, which solves all system failures: two microservices id1 and id2 deployed on that board and another microservice id3 depends on the failed id2 according to rule (5.6).

5.4 $$\begin{array}{rl} & \;\;⟹\;\;createOn(id1,d1) \end{array}$$

5.5 $$\begin{array}{rl} & \;\;⟹\;\;microservice(id1,s1) \end{array}$$

5.6 $$\begin{array}{rl} ms(X,s1)\wedge ms(Y,s2)\wedge sameRoute(X,Y) & \;\;⟹\;\;depend(Y,X) \end{array}$$

In this example, the two insufficiencies are caused by the incompleteness of the system log: the information of (5.4) was missing, and the theory is formalized from multiple data sources which caused the mismatch between (5.5) and (5.6), respectively. Then the repair of the former reminds engineers to improve the log quality by adding the MI. The repair of renaming ms to microservice contributes to aligning the knowledge from the different data sources. In addition, the repair of the second step of RCA provides the solution of fixing these system failures: ensuring that board d1 is not full.

### (d) New physics by analogy

For his 2016 MSc project, Cheng-Hao Cai applied reformation to the problem of correcting faulty analogies [13,26]. One of these faulty analogies was between gravitational attraction and electrostatic attraction/repulsion. In particular, an approximation to Coulomb’s law of electrostatic force can be generated from Newton’s law of universal gravitation

$$F=G.\frac{m_{1}.m_{2}}{r^{2}},$$

where F is the gravitational force acting between two objects of mass $m_{1}$ and $m_{2}$, r is the distance between their centres of mass and G is the gravitational constant. However, corresponding electrostatic charges repel rather than attract and G must be replaced by Coloumb’s constant $k_{e}$.

2016 predates ABC, so Cai applied just reformation. He also needed equational reasoning in addition to resolution, so adapted reformation to work with the Z3 solver [27]. The equivalents of Reformation 1s was used to change attraction to repulsion and Reformation 3s to change G to $k_{e}$.

### (e) Modelling student misconceptions

When students learn arithmetic, they may make mistakes. Jovita Tang’s 2016 MSc project used reformation to model students’ misconceptions of arithmetic procedures [28]. The preferred structure was the students’ incorrect answers to arithmetic problems T(PS), with the correct mathematical calculation rules R treated as the original faulty theory. ABC repaired R into a theory that models the student’s incorrect mathematical calculation, i.e. theory $R^{\prime }$ is a logic program that derives the student’s miscalculations in T(PS) as theorems. The repairs required to do this highlight the student’s misconceptions.

## 6. Conclusion

We have successfully evaluated our first claim that representational change is integral to the reasoning for both humans and computers. As arguments are fleshed out, faults are exposed by the reasoning process that then has to be repaired. Such repair frequently involves an elaboration of the representation on which the reasoning is based. New concepts must be defined or existing ones refined. Unexpected distinctions are required and the language of the representation is refined to attest to them. For instance, an analysis of Lakatos’ ‘Proofs and Refutations’ shows that, even in mathematical proofs, the objects of the reasoning are revealed to be vague and require elaboration.

To automate representational change, we have developed the ABC system, which combines abduction, belief revision and conceptual change (reformation) to repair faulty theories. We have successfully evaluated our second claim that the ABC system has a diverse range of successful applications to repair faulty representations. We describe diverse successful applications to defeasible reasoning, virtual bargaining, RCA, analogy repair and modelling student misconceptions. These applications include both technological and cognitive domains. It is sometimes necessary to model faults, e.g. in a 5G network or a student’s misconceptions. In these cases, ABC can be used to work backwards, from a theory of the ideal situation, via symptoms of the fault, to the faulty situation being modelled.

Lastly, the question naturally arises as to whether the examples of representational change in [1], that we discussed in §1, can be automated by the ABC system. The answer is yes, potentially. We explored this question in an unpublished note.^10^ Reformation 1c can be used to bar the three-dimensional interpretation of the hollow cube and Belief Revision 2 to bar the pentagon as a polygon. However, representing some of the concepts involved, such as a polyhedron, required existential quantification and unary functions, which takes us beyond Datalog. A full first-order version of ABC is, therefore, required, so this challenge remains for future work.

## Data Availability

The ABC system can be downloaded from GitHub at https://github.com/XuerLi/ABC_Datalog. The data are provided in the electronic supplementary material [29].

## Footnotes

1 This requirement can be relaxed providing one is willing to accept a heuristic ABC. That is, proof attempts can be terminated with failure when some time or space threshold is exceeded. So, a difficult to prove theorem might be wrongly classified as unprovable. In fact, a predecessor of ABC was originally developed for the semi-decidable FOL [7].

2 The opposite of the Prolog convention.

3 The definition of its language.

4 A black swan can be seen in St James’s Park, just opposite the Royal Society’s HQ.

5 This choice seems to work tolerably well for most examples.

6 Some logicians regard monotonicity as a *defining* property. For them, a non-monotonic logic is an oxymoron.

7 Note that, in order to stay within the Datalog grammar, ≠ is not the negation of =, but a binary predicate in its own right with the same meaning.

8 To avoid misunderstanding, ‘system’ in this subsection only refers to the object where system failures occur, rather than the ABC repair system.

9 A cause may be missing while its logical consequence exists in a theory, e.g. only the latter is recorded in the log.

10 ‘Can ABC do Lakatos-Style Monster Barring?’, Alan Bundy, Blue Book Note 1882.
